# Sleep disorders and work-related stress with oral hygiene among Indian shift workers

**DOI:** 10.6026/97320630019069

**Published:** 2023-01-01

**Authors:** Kumaresan Sathya, D Sri Sakthi, N.D Jayakumar, Arumugham Indiran Meignana

**Affiliations:** 1 Saveetha Institute of Medical and Technical Sciences, Saveetha University, Chennai, India; *Corresponding author

**Keywords:** Work ability index (WAI), Berlin questionnaire, oral hygiene index-Simplified (OHI-S), obstruction sleep apnea, construction workers, migrant workers

## Abstract

Construction workers are mostly migrants from isolated villages and are not vigilant about health care measures besides poor language skills. Majority of the population works in shifts across the globe. As a result of poor sleep architecture, excessive sleepiness or insomnia, the construction labourers working in shifts have approximately twice the risk of OSA when compared with those working in the daytime. Likewise, the performance and the productivity of employees in construction sites are impeded by added stress. Therefore, it is of interest to investigate the sleep disorders, work-related stress and its impact on oral hygiene among the construction workers in Chennai. A cross sectional study was conducted among 518 workers in various construction sites at Chennai, South India. The study incorporated BerlinQuestionnaire to evaluate disordered breathing during sleep, the Work Ability Index that contains questions concerning work, working ability and health and the Oral Hygiene Index Simplified (Greene and Vermillion, 1964) that was used for recording the oral hygiene status. Pearson correlation between education and OHI-S was statistically significant (r=-0.108). Multiple linear regression analysis revealed that mean WAI score had a positive significant association with work experience (B=0.059, SE=0.030, p=0.05), habits (B=0.032, SE=0.017, p=0.05) and marital status (B=0.135, SE=0.54, p=0.01). In contrast, education (B=-0.0.052, SE=0.023, p=0.02) and work schedule (B=0.022, SE=0.037, p=0.54) were inversely associated with the mean score. Based on the current findings, it is imperative to restore work ability for those with poor work ability thereby enhancing productivity in the migrant workers. As shift work may be extremely detrimental to poor sleep quality, the employers should arrange shift schedules in accordance with sleep physiology. Additionally, dental awareness and interventions are required to improve oral hygiene among migrant workers.

## Background:

Work is one enclosure from which most adults ensure gratification in life. It is the stereotyped stressful experience [1]. Hence, stress phenomenon is one of the intrinsic problems in recent decades that threaten human health [2]. According to the National Institute of Occupational Safety and Health, work related stress or job stress is defined as the harmful physical and emotional responses that arise when the job requirements are nconsistent with the capabilities of the worker [1,2,3]. Job stress is increasingly incriminated in the development of heart disease, mental ill health and musculoskeletal disorders besides an early retirement owing to ill health. Likewise, the performance and the productivity of employees in the organisation are impeded by added stress. Furthermore, 50-60% of all lost working days are in accord with occupational stress. Thus, improved work ability is one of the most effective ways to prevent disability and early retirement [4]. About 20% of the employed population works in shifts across the globe. Shift workers have a higher incidence of sleep problems than fixed day workers [5]. Shift work causes interruption in the sleep wake cycle due to misalignment in the endogenous circadian rhythm [6].As a result of poor sleep architecture, excessive sleepiness or insomnia, shift workers have approximately twice the risk of accidents when compared with those working in the daytime [7]. Excessive sleepiness is a common squeal of poor quality of sleep and is reported in approximately 30% of patients with Obstructive Sleep Apnoea OSA [8]. OSA is a consequence of periodic episodes of upper airway collapse during sleep leading to sleep impairment. OSA has been associated with acute sleep deprivation which is likely the catalyst for poor performance [9]. It is correlated with attention, memory and executive function impairments in regard to neurocognitive function in shift workers. Construction site workers at workplace are vulnerable to various health hazards. The tough, manual work usually in exceptional conditions, lower literacy rate, loaded working hours, lesser income and lack of access to health services make their situations unworthy [11]. They are mostly migrants from isolated villages and are not vigilant about health care measures besides poor language skills. In addition, poor literacy and low socioeconomic status have resulted in the practice of tobacco usage in them [12][13]. As there has been no such studies conducted on the construction site workers regarding the effects of sleep disorders and work-related stress. Therefore, it is of interest to investigate the sleep disorders, work-related stress and its impact on oral hygiene among the construction workers in Chennai.

## Methods:

## Study design and setting:

This cross-sectional study was carried out in various construction sites at Chennai, South India from May 20, 2022 to July 20, 2022. The study participants were recruited from routine dental camps for the migrants organized by the Department of Public Health Dentistry, Saveetha Dental College. The workers were selected through convenient sampling method. The participation in the study was voluntary without undue pressure. The study was approved by Institutional Review Board of Saveetha University. Additionally, approval was obtained from the Company's Ethics Committee. After obtaining the informed consent, the study sample consisted of a total of 518 construction site workers, including shift workers and fixed day workers in different fields. Two examiners were standardised and calibrated in the Department of Public Health Dentistry, Saveetha Dental College to ensure an uniform interpretation, understanding and application of codes used in the study.

## Inclusion and Exclusion criteria:

The workers included in the study had been working in their shift schedule for at least one year. Workers with current tobacco use were included in the study. The workers were excluded if they were not willing to participate in the survey or had a family history of sleep disorders.

## Data collection:

Data were collected using a pre validated questionnaire comprising of 4 parts as follows: Socio demographic data (age, gender, marital status, education, work experience, work schedule and habits), Assessment of oral hygiene status, Work ability questionnaire and Berlin questionnaire to assess sleeping disorders in the migrant workers. The questionnaire used was translated into local language, that is Tamil and also in Hindi, as mostly the workers were migrants, following which it was back translated.

## Variables:

The questionnaire incorporated Berlin Questionnaire to evaluate disordered breathing during sleep and the Work Ability Index that contains questions concerning work, working ability and health. The Oral Hygiene Index Simplified (Greene and Vermillion, 1964) was used for recording the oral hygiene status.

## Berlin Questionnaire:

The Berlin Questionnaire assesses risk of breathing disorders during sleep through 10 questions regarding body mass index, snoring, sleep state and blood pressure. The scale includes three separately evaluated categories: individuals with positive results in at least two of the three categories were considered at high risk for obstructive sleep apnoea syndrome.

## Work ability Questionnaire:

Work ability is defined as the ability of workers to accomplish their job, and being cognizant of work demand, physical and mental conditions. The work ability index is a prominent instrument to conceptualize work ability. The cumulative index of WAI ranges from 7 to 49 points. It is divided into categories: Poor (7-27 points), moderate (28-36 points), good (37-43 points) and excellent work ability (44-49 points). Subjects at or below 36 points were classified as having low work ability. Subjects at or above 37 points were classified as having satisfying work ability.

## Results:

## Statistical analysis:

Statistical Analysis was performed using SPSS Software Package (version 23). Normality tests like Shapiro-Wilks and Kolmogorov-Smirnov were estimated. Descriptive statistics were used to describe the characteristics of the study population. The effect of demographics variables on the Work Ability Index was investigated using Multiple Linear Regression Analysis. The Pearson Correlation used to test the association between habits, education and OHI-S. The level of statistical significance was predetermined to be p<0.05. (Table 1) summarizes the demographic details of the workers who participated in the study. Majority of the participants were male workers ranging from 20 years to 50 years. A majority of the participants had middle school education 210 (40.1%).Some 318 (60.7%) of the study population was married and 267 (51%) of the participants were rotating shift workers.

## Berlin Questionnaire Scores:

333(63.5%) of 518 participants had a high risk for OSA on the BQ,while 185 (35.3%) had a low risk score. Of 518 participants,339 (64.7%) had a positive score in Category 1 of the BQ,486(92.7%) had a positive score in Category 2 and 135 (25.8%) positive score in Category 3 of the BQ(Table 2).

## WAI Scores:

According to the WAI categorical classification, most of the participants had a good (54.6%) or excellent (37.8%) as shown in (Table 3).

## OHI-S Scores:

141 (26.9%) of the participants had good oral hygiene, 230 (43.9%) of the participants had fair oral hygiene and 147 (28.1%) of the participants had poor oral hygiene (Table 4). In order to investigate the correlation between habits, education and OHI-S index in the study participants, Pearson correlation coefficient was used. The results of this analysis showed that although the correlation between habits and OHI-S was not statistically significant (p>0.05), the correlation between education and OHI-S was statistically significant (p=0.01).This relationship was negative and the correlation coefficient (r=-0.108) indicated a weak correlation between these two variables. This means that with a decrease in the education, the mean OHI-S score would increase (Table 5). The results of Linear Regression Analysis, controlling for other significant variables such as education, marital status, work schedule, work experience and habits revealed that mean WAI score had a positive significant association with work experience (B= 0.059 , SE=0.030, p=0.05), habits (B=0.032, SE=0.017, p=0.05) and marital status (B=0.135, SE=0.54, p=0.01). In contrast, education (B=-0.0.052, SE=0.023, p=0.02) and work schedule (B=-0.022, SE=0.037, p=0.54) were inversely associated with the mean score. Marital status showed a strong association with the mean WAI score. Meanwhile, a negative and non-significant association was found between mean WAI and work schedule (Table 6).

## Discussion:

Current study was conducted among the construction workers in the various parts of Chennai district to investigate the sleepdisorders and work-related stress and its impact on oral hygiene in them. The reason to go for construction workers was that instead of being the most integral part of urbanization in the city, they are the victims of occupational hazards involving tough manual labour, low literacy rate and poverty in conjunction with ignorance. A total of 518 workers were interviewed and examined. The majority of the migrants belonged to the age group of 40-50 years.23.1% of the total workers belonged to the age group of 30-40 years. Among the tobacco users, 125 used the smoked form, 139 used the smokeless form and 171 used both. The study shows that there is a high incidence of tobacco consumption among the migrants. An increase in the number and the chronicity of oral habits among the migrant workers has serious health repercussions and can intensify the incidence of tobacco related oral cancer in them [14, 15]. In our study, Work ability assessed by WAI found that 34 workers had moderate work ability and 286 workers had good work ability. In a study conducted in Norway on work ability among female employees, 8.9% reported an extremely or very reduced ability to work [16]. Work ability was found to be significantly associated with education, marital status, work experience and habits. A study conducted in plantation workers in South India found an association of work ability with marital status and work experience similar to our studies. There is a negative correlation between Work ability and BMI [16, 17]. Similarly, our study showed a negative and non-significant association between mean WAI and work schedule. Deprived sleep architecture in shift workers has been emphasized previously in the literature [18-21]. In a study conducted by Thach et al. shift work was significantly associated with increased odds of poor sleep quality in their workers (18). Previous polysomnography studies validated that night shifts exacerbated the symptoms of OSA [22-23]. However in our study, we did not conduct polysomnography. In our study, 333 (63.5%) of 518 participants had a high risk for OSA on the BQ, while 185 (35.3%) had a low risk score. These findings are consistent with previous studies reporting a higher prevalence of OSA among shift workers compared with general population [24]. Smoking, alcohol use and gastrointestinal diseases were distinguished as risk factors for poorer sleep quality which supports the literature [25] [26]. Findings of the present study indicate that 141 (26.9%) of the participants had good oral hygiene, 230 (43.9%) of the participants had fair oral hygiene and 147 (28.1%) of the participants had poor oral hygiene. Akanksha et al reported similar results in which 86.67% of workers maintained their oral hygiene and 60% of the study population had shown a poor oral hygiene [27]. The poor oral hygiene status could be accountable to the lack of awareness of proper brushing techniques. This emphasizes the need to maintain good oral hygiene among the migrant workers [28].

## Limitations of the study:

The convenient sampling of the study participants may affect the generalization of results of the study. In addition, there are chances of respondent's bias. The presence of collateral diseases in our study was assessed through the participant's self-reports and was not confirmed by laboratory tests.

## Conclusion:

Based on the current findings, it is imperative to restore work ability for those with poor work ability, arrange work schedules in accordance with sleep physiology and health promotion activities directed towards alcohol and tobacco cessation as construction site workers are in need of high oral care and it is essential to address the gap between the need for dental care and the amount of dental care received. Moreover, dental awareness and interventions are required to improve oral hygiene among migrant workers. In addition, the employers should regulate the working hours as shift work may be extremely detrimental to poor quality of sleep. Work ability and productivity in the migrant workers can be enhanced by periodic health examinations to attenuate musculoskeletal problems, regulation of BMI or other screening procedures.

## Figures and Tables

**Table 1:** Demographic data of the participants

Age	Frequency	Percentage
Less than 20 years	46	8.8
20-30 years	111	21
30-40 years	121	23.1
40-50 years	220	42
Gender		
Male	512	98.9
Female	6	1.1
Education		
Illiterate	181	34.5
Middle school	210	40.1
High school	126	24
Undergraduate	1	2
Marital status		
Married	318	60.7
Unmarried	200	38.2
Work schedule		
Fixed night	105	20
Rotating shift	267	51
Fixed day	146	27.9
Work experience		
11-20 years	264	50.4
21-30 years	96	18.3
31-40 years	158	30.3
Habits		
Smoking alone	125	23.9
Alcohol	83	15.8
Betel nut	139	26.5
Smoke and smokeless	171	32.6

**Table 2:** Berlin Questionnaire Scores

	Frequency	Percentage
High Risk	333	63.5
Low Risk	185	35.3

**Table 3:** Work ability among construction workers

	Frequency	Percentage
Moderate	34	6.5
Good	286	54.6
Excellent	198	37.8

**Table 4:** OHI-S

	Frequency	Percentage
Good	141	26.9
Fair	230	43.9
Poor	147	28.1

**Table 5:** Correlation between Habits, Education and OHI-S using Pearson's Correlation

Variables	Pearson's correlation value	P value
Habits and OHI-S	0.019	0.06
Education and OHI-S	-0.108	0.01

**Table 6:** Multiple Linear Regression analysis results for WAI scores by demographic variables

Demographic Variables		Unstandardized coefficients	Standardized coefficients	p value
	B	Std. Error		
Education	-0.052	0.023	-0.1	0.023
Marital status	0.135	0.54	0.112	0.012
Work schedule	-0.022	0.037	-0.026	0.548
Work experience	0.059	0.03	0.088	0.050
Habits	0.032	0.017	0.086	0.052
